# Stepwise nutritional management combined with multimodal rehabilitation for severe dysphagia following medullary infarction in an elderly patient: a case report

**DOI:** 10.3389/fnut.2026.1788301

**Published:** 2026-06-01

**Authors:** Dandan Cheng, Yan Chen, Yiwen Li, Hua Shen, Shujin He, Ye Chen, Qianru Chen, Huiwen Mao, Yan Li

**Affiliations:** Rehabilitation Department, Tongren Hospital, Shanghai Jiao Tong University School of Medicine, Shanghai, China

**Keywords:** dysphagia, medullary infarction, multidisciplinary team (MDT), neuromodulation, nutritional management

## Abstract

Nutritional support and rehabilitative training are crucial for functional recovery in stroke patients with dysphagia. Malnutrition often aggravates motor, cognitive, and emotional deficits, underscoring the need for tailored nutritional strategies to improve swallowing and motor outcomes. We present a case of severe dysphagia complicated by secondary gastroparesis following a medullary infarction. After assessment by a multidisciplinary team (MDT), a multimodal rehabilitation protocol was implemented, consisting of repetitive transcranial magnetic stimulation (rTMS), transcranial direct current stimulation (tDCS), and neuromuscular electrical stimulation (NMES), combined with conventional swallowing training. Concurrently, a structured stepwise nutritional management plan was executed, progressing from nasojejunal (NJ) feeding to intermittent oro-esophageal (IOE) tube feeding, and finally to independent oral intake. After 3 months of integrated treatment, the patient’s Modified Mann Assessment of Swallowing Ability (MMASA) score improved from 81 to 94(out of 100). Videofluoroscopic Swallowing Study (VFSS) demonstrated a reduction in laryngeal elevation latency and safe pharyngeal transit, allowing for the successful removal of the feeding tube. This case suggests that MDT-guided stepwise nutritional management, when combined with multimodal swallowing rehabilitation, may offer a beneficial and feasible therapeutic approach for severe dysphagia post-medullary infarction. This integrated model successfully balances therapeutic safety with functional recovery, providing a practical framework for breaking the vicious cycle of post-stroke dysphagia and malnutrition.

## 1 Introduction

Stroke remains a leading cause of global mortality and long-term disability, with post-stroke dysphagia (PSD) appearing in 37%–78% of survivors (1). PSD is not merely a mechanical dysfunction but a complex physiological state that significantly increases the risk of aspiration pneumonia, malnutrition, and a 2.3-fold increase in three-month mortality (2). This burden is exceptionally severe in medullary infarction, where damage to the brainstem’s central swallowing networks—including the nucleus tractus solitarius and nucleus ambiguus—disrupts the fundamental sensorimotor coordination required for safe oral intake (3). Beyond immediate feeding difficulties, PSD initiates a “malnutrition-inflammation” cycle; stroke-induced malnutrition triggers systemic immune dysregulation and inflammation, which in turn impairs neuroplasticity and delays functional recovery (4). Effective management of severe PSD requires an integrated paradigm that moves beyond passive feeding to active neural and metabolic rehabilitation. Neuromodulation techniques have emerged as powerful tools to drive this recovery by facilitating cortical reorganization (5). Specifically, techniques such as repetitive transcranial magnetic stimulation (rTMS) and transcranial direct current stimulation (tDCS) modulate cortical excitability, rebalancing interhemispheric inhibition and inducing neuroplasticity in the pharyngeal motor cortex (6, 7). Complementarily, NMES bridges central and peripheral pathways through suprahyoid recruitment and sensory feedback, synergizing with conventional therapy to prevent disuse atrophy (8). The synergistic efficacy of these combined modalities has been increasingly validated in acute post-stroke dysphagia (9). In parallel, the nutritional delivery route itself serves as a critical determinant of clinical outcomes. While nasogastric (NG) tubes are standard, they often exacerbate gastroparesis and gastroesophageal reflux in elderly patients, potentially fueling aspiration-mediated pulmonary inflammation and further impairing the swallowing reflex (10). Current international guidelines emphasize that early and individualized enteral support is pivotal for preventing catabolism and supporting neuroplasticity (ESPEN practical guideline) (18). Recent clinical evidence suggests that transitioning to advanced enteral access, such as NJ feeding for gastric dysmotility, followed by IOE tube feeding, can minimize airway secretions and promote functional recovery of the swallowing reflex compared to continuous feeding (11).

Here, we report the case of an elderly patient with medullary infarction and severe dysphagia complicated by secondary gastroparesis. By implementing an MDT-guided model that integrated high-focality neuromodulation with a “Monitor-Adapt-Upgrade” stepwise nutritional plan, we successfully navigated the transition from NJ-dependent feeding to independent oral intake.

## 2 Case description

An 83-year-old female presented with a 2-week history of dysphagia and left-sided weakness. Brain MRI confirmed an acute right medullary infarction with multiple lacunar lesions in the basal ganglia. Despite initial antiplatelet and neuroprotective therapy, her clinical course was quickly complicated by aspiration pneumonia (peak temperature 39.2 °C). Neurological examination revealed dysarthria, hoarseness, mild left facial paresis, and facial numbness. Motor assessment showed left hemiparesis (Medical Research Council [MRC] grade 4 in the upper limb, 3 in the lower limb). Swallowing and laryngeal evaluations indicated restricted tongue mobility without atrophy, diminished laryngeal elevation, severely reduced maximum phonation time (5 s), and delayed pharyngeal and gag reflexes, although voluntary cough remained intact. Initial nasogastric (NG) feeding was poorly tolerated due to persistent nausea, vomiting, and gastroesophageal reflux, strongly suggesting secondary gastroparesis.

**Figure 1:**
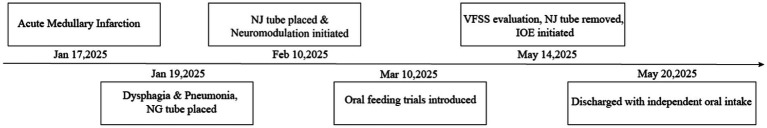
Clinical timeline of the patient’s disease progression, swallowing assessments, and stepwise nutritional interventions.

**Figure 2:**
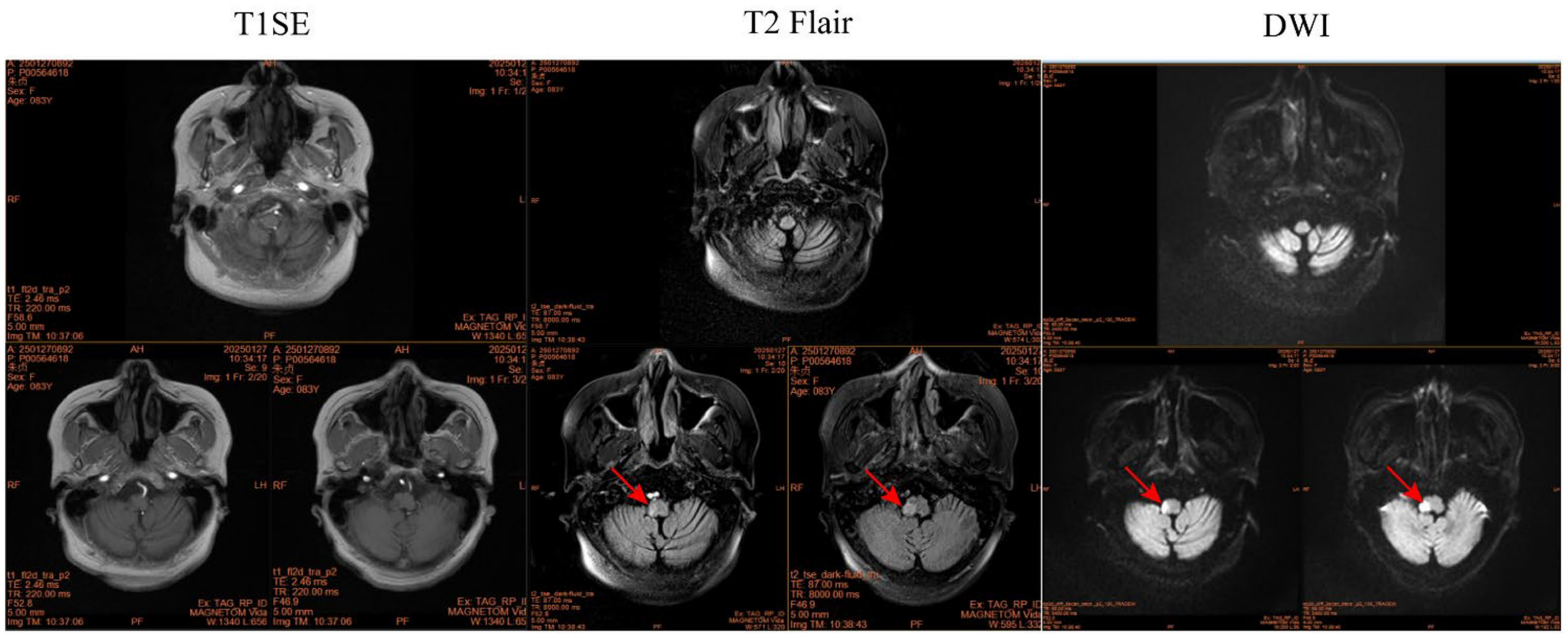
Head MRI on February 9 showed a right medulla oblongata subacute infarction. Axial diffusion-weighted imaging (DWI) sequence shows high signal intensity in the right medulla oblongata (arrow), indicative of acute infarction. Axial fluid-attenuated inversion recovery (FLAIR) sequence reveals multiple lacunar lesions in the bilateral basal ganglia (arrowheads).

Admission laboratory findings highlighted protein malnutrition (albumin 30 g/L and hemoglobin 102 g/L) despite a high-normal baseline BMI (height 150 cm, weight 57 kg, BMI 25.3 kg/m^2^). Her medical history was notable for poorly controlled hypertension and type 2 diabetes (HbA1c 6.6%) managed with oral medications. The clinical timeline and representative neuroimaging findings are summarized in Figure 1 and Figure 2, respectively.

### 2.1 Nutritional and swallowing functional assessment

Nutritional Risk Screening (NRS-2002) yielded a score of 5, indicating high nutritional risk. Standardized swallowing assessments yielded a Modified Mann Assessment of Swallowing Ability (MMASA) score of 81 (out of a maximum score of 100) and a Kubota Water Swallowing Test grade of 5, confirming severe dysphagia. The Volume-Viscosity Swallowing Test (V-VST) indicated that the patient could only safely tolerate 3 mL of pudding-consistency food; critically, even 3 mL of thin liquid triggered immediate coughing and oxygen desaturation (SpO2 < 90%). This explicit indication of a severely impaired laryngeal vestibule closure drove our clinical reasoning to strictly contraindicate oral feeding and immediately establish a safe enteral route.

### 2.2 Swallowing and nutritional therapy

To address the patient’s complex condition, we implemented a multidisciplinary team (MDT) framework to seamlessly integrate neuro-rehabilitation, metabolic optimization, and gastroenterological monitoring (Table 1). The patient’s initial MMASA score of 81 highlighted an extreme risk of silent aspiration. Compounded by clinical signs of secondary gastroparesis—namely frequent regurgitation, vomiting, and abdominal distension—continued nasogastric (NG) feeding posed an unacceptable risk of retrograde aspiration. Prioritizing airway safety, the MDT promptly transitioned the patient to endoscopic nasojejunal (NJ) feeding. By anatomically bypassing the dysmotile stomach, this approach established a vital barrier against aspiration pneumonia during the acute phase.

**Table 1:** Stepwise multidisciplinary management of nutritional support and swallowing rehabilitation.

Stage (timeline)	Functional assessment and rationale	Nutritional intervention and targets	Swallowing and neuromotor rehabilitation	Delivery method and rate
Stage I: acute phase (Weeks 1–3)	MMASA: 81 (severe risk of silent aspiration). V-VST: Desaturation with 3 mL thin liquid. Gastric: secondary gastroparesis and reflux.	Target: 25 kcal/kg/d; protein 1.2 g/kg/d. Formula: low-fat short-peptide formula (0.9 kcal/mL).	Airway protection: strict NPO (nil per os). Oral hygiene, 30° head elevation, and passive range-of-motion exercises.	NJ continuous infusion: Initiated at 30 mL/h, progressively titrated to 90 mL/h.
Stage II: stabilization (Weeks 4–7)	Gastric: reflux ceased; gastroparesis resolved. Metabolic: stable glycemic control (<10 mmol/L).	Maintenance: target caloric intake met. Formula: transitioned to standard high-energy formula (1.2 kcal/mL).	Sensory activation and neuromodulation (from Week 4) Thermal-tactile stimulation, sensory input (ice/sour), and passive lingual stretching. • rTMS: 5 Hz, 100% RMT, 750 pulses/session. • tDCS: 1.5 mA, 20 min. • NMES: 250 μs, 80 Hz. Therapeutic feeding training (from week 6) Behavioral: lingual resistance and shaker exercise. High-intensity behavioral: • sokinetic/isometric shaker regimen (3 sets/day). • Chin tuck against resistance (CTAR), expiratory muscle strength training.	NJ continuous infusion: Maintained at 90 mL/h via enteral pump.
Stage III: transition (Weeks 8–16)	MMASA: >90 (improved). V-VST: safe tolerance of 5 mL medium-thick puree. Motor: Brunnstrom Stage IV.	Diet: oral trials introduced (5–10 mL medium-thick purees). Target: protein increased to 1.5 g/kg/d (leucine-fortified whey protein).		Oral-tube hybrid: Cyclic NJ feeding (10–14 h/day) to encourage daytime oral functional drive.
Stage IV: rehabilitation (Week 17 and discharge)	VFSS: improved cricopharyngeal opening; minimal pharyngeal residue.	Diet: transitioned to oral soft solids. Supplement: oral nutritional supplements (ONS) for hydration and caloric bridge.		NJ removed; IOE initiated: Intermittent IOE bolus (3–4 sessions/day) + independent oral intake.

#### 2.2.1 Acute phase: Nasojejunal (NJ) tube feeding

Following the endoscopic placement of the NJ tube in Week 1, a targeted nutritional protocol was initiated. Given the patient’s high nutritional risk (NRS-2002 score of 5), daily caloric and protein targets were established at 25 kcal/kg (approximately 1,500 kcal/d) and 1.2 g/kg, respectively, in accordance with ESPEN guidelines. Enteral nutrition was delivered using Fresubin Diabetes—a low-fat, short-peptide formula (0.9 kcaL/mL)—via a continuous pump at a rate of 90 mL/h. To further support gastrointestinal motility and metabolic homeostasis, prokinetic agents (itopride) were administered, and blood glucose levels were strictly maintained below 10 mmol/L. By Week 4, the continuous NJ feeding was well-tolerated, with complete cessation of gastroesophageal reflux.

#### 2.2.2 Stabilization and transition period: multimodal rehab and oral-tube hybrid training

##### 2.2.2.1 Multimodal rehabilitation (initiated in week 4)

To facilitate cortical reorganization early in the stabilization phase, a structured neuromodulation protocol was executed concurrently with NJ feeding. Central stimulation included 5 Hz rTMS (100% RMT, 750 pulses/session, 5 sessions/week) targeting bilateral M1 oropharyngeal areas, and tDCS (1.5 mA, 20 min; anode over M1 orolingual region, cathode over the contralateral supraorbital area). Peripherally, NMES (250 μs, 80 Hz) was applied to the suprahyoid muscles. This integrated “top-down” and “bottom-up” approach was synergized from the outset with high-intensity behavioral interventions, including Shaker training (a supine head-lift regimen targeting the suprahyoid and infrahyoid musculature), Chin Tuck Against Resistance (CTAR: neck flexion against a submandibular resistance device to specifically recruit the suprahyoid muscles), and sensory stimulation. All rehabilitative modalities were continuously maintained throughout the patient’s recovery trajectory.

##### 2.2.2.2 Therapeutic and functional feeding transition

Starting Week 6, once the MMASA score exceeded 90 and the V-VST confirmed safe tolerance of 5 mL medium-thick puree, the patient initiated therapeutic oral feeding training. At this stage, small-volume oral boluses (5 mL) were utilized purely as a rehabilitative stimulus to re-engage neural swallowing pathways, rather than for nutritional intake. This assessment-driven strategy proactively restored neuromuscular reflexes while maintaining a strict safety margin against aspiration. Bolus volumes were progressively titrated (e.g., from 5 mL to 10 mL) only after consecutive safe swallows, strictly defined by the absence of clinical aspiration signs (coughing/wet voice) and stable SpO2 (≥95%).

By Week 8 (March 10, as shown in Figure 1), as swallowing coordination substantially improved, the patient progressed to a functional oral-tube hybrid feeding model. Oral intake began actively contributing to her daily nutritional requirements, supplemented by cyclic NJ feeding. Concurrently, to address post-stroke sarcopenia as limb mobility progressed (Brunnstrom Stage IV), daily protein intake was increased to 1.5 g/kg utilizing leucine-fortified whey protein.

#### 2.2.3 Rehabilitation phase: VFSS-guided transition and IOE implementation

By Week 12, with gastroparesis fully resolved (gastric residual volume <100 mL) and the MMASA score reaching 94, a repeat VFSS revealed significantly improved cricopharyngeal opening and reduced pharyngeal residue. Consequently, the MDT removed the indwelling NJ tube and initiated intermittent oro-esophageal (IOE) tube feeding.

Implemented as a critical transition strategy, IOE involves the temporary insertion of a feeding tube for each bolus delivery. Unlike indwelling tubes, this repeated mechanical action provides potent sensorimotor stimulation to the pharynx and esophageal sphincters, facilitating swallowing reflex coordination and preventing disuse atrophy. Furthermore, IOE safely bridged the patient’s hydration and caloric needs (500–800 mL/day) while preserving her functional drive for independent oral intake of soft solids between feedings.

**Figure 3:**
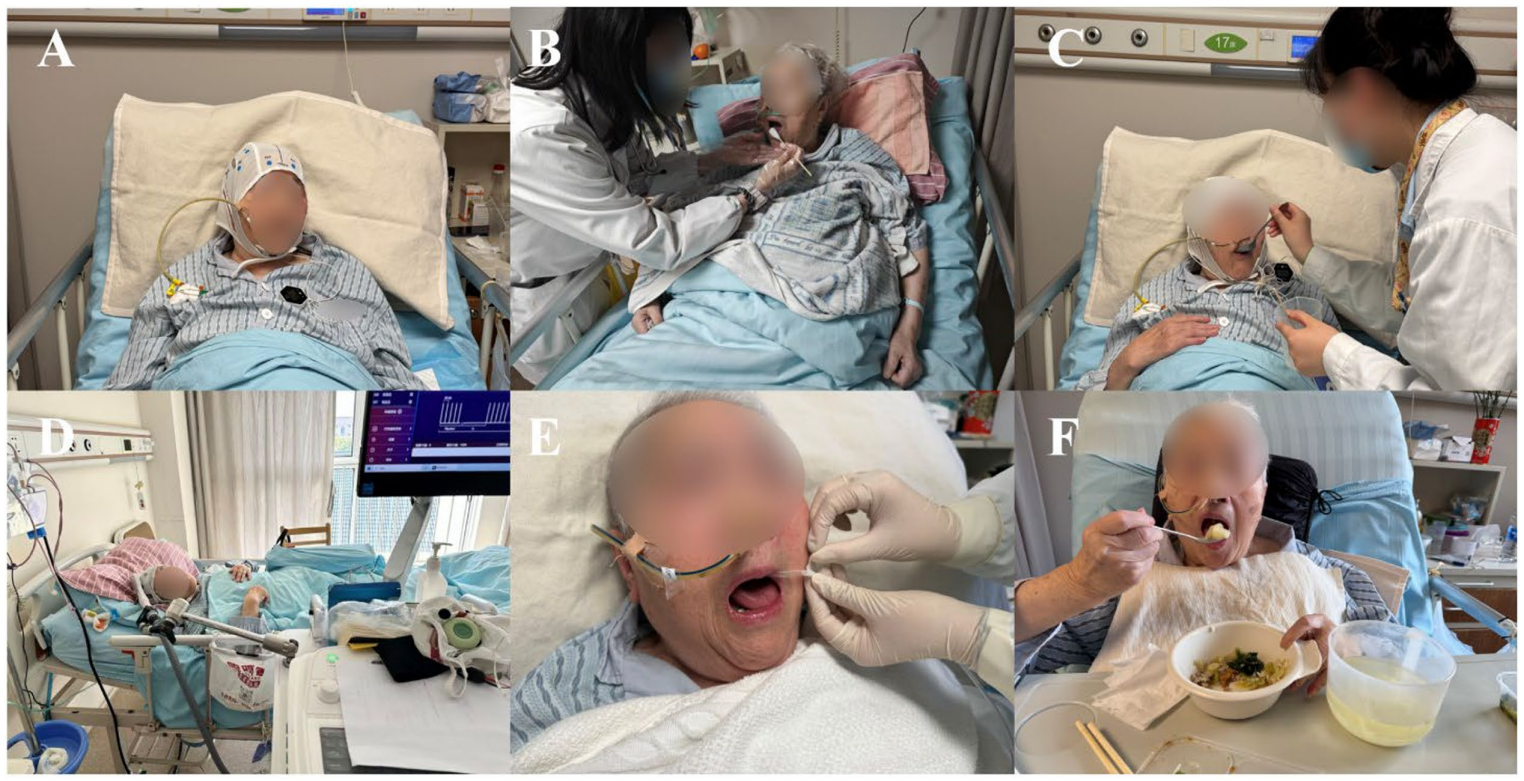
Multimodal rehabilitation interventions and nutritional management. **(A)** Transcranial direct current stimulation (tDCS) targeting swallowing motor cortex. **(B)** Sensory stimulation combined with oromotor exercises. Note: Ice stimulation and manual facilitation are shown. **(C)** Repetitive transcranial magnetic stimulation (rTMS) targeting swallowing motor cortex. **(D)** Therapeutic feeding training assisted by a therapist. **(E)** Intermittent oro-esophageal (IOE) tube feeding procedure. **(F)** Patient performing self-feeding training with modified diet consistency.

Ultimately, nutritional markers improved significantly (weight stabilized at 60 kg, albumin 35 g/L, hemoglobin 109 g/L), and motor function advanced to Brunnstrom Stage V. The patient was successfully discharged on an oral soft diet supplemented by family-led IOE, with no feeding-related complications (Figure 3).

### 2.3 Outcomes

By May 19, the MMASA score had improved from 81 to 94 (near-normal range). VFSS confirmed significant improvement in laryngeal elevation and successful clearance of residues through repetitive swallowing. Nutritional markers improved significantly: weight increased to 60 kg, albumin rose to 35 g/L, and hemoglobin reached 109 g/L. Motor function also progressed to Brunnstrom Stage V, and the Modified Barthel Index rose from 20 to 50. The patient was discharged successfully using oral soft foods supplemented by family-led IOE feeding, with no feeding-related complications. Detailed rehabilitation therapies and nutritional management are presented (Figure 3 and Table 1).

## 3 Discussion

### 3.1 Pathophysiology and the multidisciplinary approach

Brainstem stroke is a predominant risk factor for the development of dysphagia (12). The brainstem houses critical nuclei governing the swallowing reflex; damage to these central structures disrupts neuromuscular control, leading to severe dysphagia, aspiration, and subsequent pneumonia. In this case, the clinical complexity was compounded by secondary gastroparesis, immobilization-induced muscle atrophy, and deep vein thrombosis risk.

Recent literature emphasizes the bidirectional relationship between metabolic homeostasis and post-stroke neuroplasticity, suggesting that chronic malnutrition not only exacerbates physical frailty but actively impedes the cortical reorganization necessary for functional recovery (13). Given that optimal nutritional status is the cornerstone of rehabilitation, we adopted a deep-collaboration MDT model. This “closed-loop” management system leveraged specialized expertise to overcome the limitations of a single-discipline perspective, ensuring the adequate provision of nutritional substrates required for neural repair (14).

### 3.2 Rationale for nutritional access: the choice of Nasojejunal feeding

Selecting an appropriate enteral feeding route is a critical decision that must balance nutritional safety, metabolic adequacy, and patient-centered care. Although NG tubes remain the first-line choice for short-term feeding due to ease of placement, accumulating evidence highlights their limitations. Prolonged NG feeding in patients with secondary gastroparesis is associated with high rates of tube-related complications and aspiration pneumonia (15). Conversely, while percutaneous endoscopic gastrostomy (PEG) is widely recommended when prolonged enteral feeding (>4 weeks) is anticipated, it is an invasive procedure. Recent evidence challenges routine early PEG placement, noting it may be considered overtreatment during the acute phase when the functional recovery trajectory remains uncertain (16).

In our case, NJ feeding was selected due to severe NG tube intolerance, uncertain duration of enteral needs, and family preference for a minimally invasive strategy. By completely bypassing the stomach, the NJ route acted as a safe metabolic “bridge” to secure hypermetabolic demands and prevent reflux-associated microaspiration—a benefit consistent with evidence that NJ feeding significantly lowers post-stroke pneumonia risk (17). Upon meeting objective clinical milestones, we transitioned to IOE feeding. Serving as a “physiological trainer,” IOE facilitated swallowing reflex recovery through periodic sensorimotor stimulation while avoiding the complications of persistent nasopharyngeal irritation (11). This dynamic adaptation of feeding routes—driven by metabolic and functional metrics rather than rigid conventional timelines—safely bridged the gap between tube dependency and full oral autonomy.

### 3.3 Limitations and future directions

This study has inherent limitations. First, as a single case report, the findings are fundamentally exploratory, and clinical decisions (e.g., timing of NJ placement) were heavily influenced by specific patient tolerance and family preferences, which may limit generalizability. Second, given the multimodal nature of the intervention (integrating stepwise nutritional transitions, rTMS, tDCS, NMES, and conventional therapy), it is intrinsically difficult to disentangle the specific therapeutic weight of each component. The observed functional recovery likely reflects the synergistic effect of these combined therapies. Finally, the lack of long-term follow-up precludes definitive conclusions regarding the durability of these outcomes. Future research should investigate adaptive decision-support tools that combine functional metrics, lab markers, and feeding tolerance to optimize stepwise nutritional rehabilitation in broader cohorts.

## 4 Conclusion

This case suggests that a stepwise nutritional management strategy, when carefully integrated within a multimodal rehabilitation program, may offer a practical and beneficial approach for managing severe neurogenic dysphagia following medullary infarction in elderly patients. Guided by a structured decision-making framework, this model successfully navigated the critical balance between patient safety, metabolic demands, and care continuity. While our findings serve as an exploratory framework rather than a definitive blueprint, this approach highlights the importance of reconciling acute hypermetabolic demands with long-term swallowing rehabilitation. Further well-designed, large-scale clinical trials are necessary to validate these exploratory findings and establish robust evidence-based guidelines.

## 5 Patient perspective

I am happy that treatments of nutritional support and rehabilitative training helped me discharged successfully. My swallowing function have become better than before. I hope that more patients who suffer from post-stroke dysphagia like me can also get a chance to these treatments.

## Data Availability

The datasets presented in this article are not readily available because of ethical and privacy restrictions. Requests to access the datasets should be directed to the corresponding authors.

## References

[1] GBD 2019 Stroke Collaborators. Global, regional, and national burden of stroke and its risk factors, 1990-2019: a systematic analysis for the global burden of disease study 2019. Lancet Neurol. (2021) 20:795–820. doi: 10.1016/S1474-4422(21)00252-0, 34487721 PMC8443449

[2] FOOD Trial Collaboration. Poor nutritional status on admission predicts poor outcomes after stroke: observational data from the FOOD trial. Stroke. (2003) 34:1450–6. doi: 10.1161/01.STR.0000074037.49197.8C, 12750536

[3] Jang SH Kim MS. Dysphagia in lateral medullary syndrome: a narrative review. Dysphagia. (2021) 36:329–38. doi: 10.1007/s00455-020-10158-3, 32654058

[4] National Alliance for Infusion Therapy, American Society for Parenteral and Enteral Nutrition Public Policy Committee and Board of Directors. Disease-related malnutrition and enteral nutrition therapy: a significant problem with a cost-effective solution. Nutr Clin Pract. (2010) 25:548–54. doi: 10.1177/0884533610378524, 20802144

[5] Labeit B Michou E Trapl-Grundschober M Suntrup-Krueger S Muhle P Bath PM . Dysphagia after stroke: research advances in treatment interventions. Lancet Neurol. (2024) 23:418–28. doi: 10.1016/S1474-4422(24)00053-X, 38508837

[6] Yang W Cao X Zhang X Wang X Li X Huai Y. The effect of repetitive transcranial magnetic stimulation on dysphagia after stroke: a systematic review and meta-analysis. Front Neurosci. (2021) 15:769848. doi: 10.3389/fnins.2021.769848, 34867171 PMC8634594

[7] He K Wu L Huang Y Chen Q Qiu B Liang K . Efficacy and safety of transcranial direct current stimulation on post-stroke dysphagia: a systematic review and meta-analysis. J Clin Med. (2022) 11:2297. doi: 10.3390/jcm11092297, 35566421 PMC9102865

[8] Wang Y Xu L Wang L Jiang M Zhao L. Effects of transcutaneous neuromuscular electrical stimulation on post-stroke dysphagia: a systematic review and meta-analysis. Front Neurol. (2023) 14:1163045. doi: 10.3389/fneur.2023.1163045, 37228409 PMC10203701

[9] Bengisu S Demir N Krespi Y. Effectiveness of conventional dysphagia therapy (CDT), neuromuscular electrical stimulation (NMES), and transcranial direct current stimulation (tDCS) in acute post-stroke dysphagia: a comparative evaluation. Dysphagia. (2024) 39:77–91. doi: 10.1007/s00455-023-10595-w, 37247074

[10] Wang ZY Chen JM Ni GX. Effect of an indwelling nasogastric tube on swallowing function in elderly post-stroke dysphagia patients with long-term nasal feeding. BMC Neurol. (2019) 19:83. doi: 10.1186/s12883-019-1314-6, 31043159 PMC6495564

[11] Juan W Zhen H Yan-Ying F Hui-Xian Y Tao Z Pei-Fen G . A comparative study of two tube feeding methods in patients with dysphagia after stroke: a randomized controlled trial. J Stroke Cerebrovasc Dis. (2020) 29:104602. doi: 10.1016/j.jstrokecerebrovasdis.2019.104602, 31924485

[12] Ko N Lee HH Sohn MK Kim DY Shin YI Oh GJ . Status of dysphagia after ischemic stroke: a Korean nationwide study. Arch Phys Med Rehabil. (2021) 102:2343–2352.e3. doi: 10.1016/j.apmr.2021.07.788, 34348122

[13] Ciancarelli I Morone G Iosa M Cerasa A Calabrò RS Tozzi Ciancarelli MG. Neuronutrition and its impact on post-stroke neurorehabilitation: modulating plasticity through diet. Nutrients. (2024) 16:3705. doi: 10.3390/nu16213705, 39519537 PMC11547614

[14] Bu F Zhang Z Qi S Xu L. From diet to brain repair: natural bioactive compounds in post-ischemic stroke recovery. Front Nutr. (2026) 13:1778396. doi: 10.3389/fnut.2026.1778396, 41971383 PMC13065517

[15] Rabaut J Thirugnanachandran T Singhal S Martin J Iievliev S Ma H . Clinical outcomes and patient safety of nasogastric tube in acute stroke patients. Dysphagia. (2022) 37:1732–9. doi: 10.1007/s00455-022-10437-1, 35296916 PMC9643179

[16] Al-Salihi MM Gillani SA Saha R Abd Elazim A Al-Jebur MS Dalal SS . Outcomes of stroke patients undergoing percutaneous endoscopic gastrostomy: a systematic review and meta-analysis. Top Stroke Rehabil. (2025) 32:294–306. doi: 10.1080/10749357.2024.2392441, 39190711

[17] Wang S Zeng X Zhang Q Li H. Effectiveness of different feeding techniques for post-stroke dysphagia: an updated systematic review and meta-analysis. Intensive Care Res. (2022) 2:108–16. doi: 10.1007/s44231-022-00022-3

[18] Burgos R Bretón I Cereda E Desport JC Dziewas R Genton L . ESPEN guideline clinical nutrition in neurology. Clin Nutr. (2018) 37:354–96. doi: 10.1016/j.clnu.2017.09.003, 29274834

