# Supplementary material for: Stepwise nutritional management combined with multimodal rehabilitation for severe dysphagia following medullary infarction in an elderly patient: a case report
Source: Front Nutr. 2026 Jun 1;13:1788301. doi: 10.3389/fnut.2026.1788301 (PMC13265362; doi:10.3389/fnut.2026.1788301)
Supplement: Supplementary file 1 [file Data_Sheet_1.pdf]

CARE case report guidelines

CARE Checklist of information to include when writing a case report

(cc) BY-NC-ND

Topic	Item	Checklist item description	Reported on Line
**Title**	1	The diagnosis or intervention of primary focus followed by the words "case report"	Title
**Key Words**	2	2 to 5 key words that identify diagnoses or interventions in this case report, including "case report"	Keywords
**Abstract (no references)**	3a	Introduction: What is unique about this case and what does it add to the scientific literature?	Abstract
	3b	Main symptoms and/or important clinical findings	Abstract
	3c	The main diagnoses, therapeutic interventions, and outcomes	Abstract
	3d	Conclusion—What is the main "take-away" lesson(s) from this case?	Abstract
**Introduction**	4	One or two paragraphs summarizing why this case is unique (**may include references**)	Case Description
**Patient Information**	5a	De-identified patient specific information.	Case Description
	5b	Primary concerns and symptoms of the patient.	Case Description
	5c	Medical, family, and psycho-social history including relevant genetic information	Case Description
	5d	Relevant past interventions with outcomes	Not Applicable
**Clinical Findings**	6	Describe significant physical examination (PE) and important clinical findings.	Case Description 2.1
**Timeline**	7	Historical and current information from this episode of care organized as a timeline	Fig1,Tab 1
**Diagnostic Assessment**	8a	Diagnostic testing (such as PE, laboratory testing, imaging, surveys).	Case Description2.1 Fig2
	8b	Diagnostic challenges (such as access to testing, financial, or cultural)	Not Applicable
	8c	Diagnosis (including other diagnoses considered)	Case Description
	8d	Prognosis (such as staging in oncology) where applicable	Line 102-108,111-120, Tab 1
**Therapeutic Intervention**	9a	Types of therapeutic intervention (such as pharmacologic, surgical, preventive, self-care)	2.2 Fig3 Tab 1
	9b	Administration of therapeutic intervention (such as dosage, strength, duration)	2.2 Tab1
	9c	Changes in therapeutic intervention (with rationale)	2.2.1-2.2.3
**Follow-up and Outcomes**	10a	Clinician and patient-assessed outcomes (if available)	2.3 Outcomes
	10b	Important follow-up diagnostic and other test results	2.3 Outcomes
	10c	Intervention adherence and tolerability (How was this assessed?)	2.2，2.3 Outcomes
	10d	Adverse and unanticipated events	Not Applicable
**Discussion**	11a	A scientific discussion of the strengths AND limitations associated with this case report	3.3 Limitations and FutureDirections
	11b	Discussion of the relevant medical literature **with references**.	3.1-3.2
	11c	The scientific rationale for any conclusions (including assessment of possible causes)	3.1-3.2
	11d	The primary "take-away" lessons of this case report (without references) in a one paragraph conclusion	4 Conclusion
**Patient Perspective**	12	The patient should share their perspective in one to two paragraphs on the treatment(s) they received	5 Patient perspective
**Informed Consent**	13	Did the patient give informed consent? Please provide if requested	Yes ☑ No ☐
